# Protocol for a scoping review of age-related health conditions among geriatric populations in sub-Saharan Africa

**DOI:** 10.1186/s13643-019-1055-z

**Published:** 2019-06-07

**Authors:** Keshena Naidoo, Jacqueline van Wyk

**Affiliations:** 1 Discipline of Family Medicine, College of Health Sciences, University of KwaZulu-Natal, Durban, South Africa; 2 Discipline of Clinical and Professional practice, College of Health Sciences, University of KwaZulu-Natal, Durban, South Africa

## Background

Sub-Saharan Africa has the most rapidly growing older population compared to any other region in the world [1]. Although the chronological age of 65 years is used to define geriatric populations in high-income countries, the United Nations agreed to the use of age 60 years to refer to geriatric populations in Africa [2]. In this scoping review, “older adults” or “geriatric population” refers to those aged 60 years and above. The geriatric population in sub-Saharan Africa (SSA) is predicted to increase from 42.6 million in 2010 to 160 million in 2050 [1]. Healthcare services for this population are delivered predominantly through the public health system at primary care level [3]. Despite the expected increase in geriatric patients that will require primary care, most primary care providers in SSA receive little to no training on geriatrics [4]. Very few countries in SSA have specialist geriatricians and there is little inclusion of geriatrics in medical curricula [5]. As a result, there has been little awareness of age-related health conditions in older adults. Most of the health care responses to geriatric health needs are based on evidence collected from populations in the high-income countries (HIC).

Advancing age is associated with physiological decline and increased risk for non-communicable diseases [6]. Geriatric syndromes, such as falls, frailty, dementia and incontinence, are complex age-related conditions that are associated with significant morbidity and poor outcomes [7]. Dementia is particularly problematic as it frequently results in disabilities and care-dependencies [8]. These age-related health conditions increase health costs and adversely affect the quality of life. The management and outcomes of health conditions in the aged are influenced by multiple factors, such as environment and management of co-morbidities [9]. People in SSA are subject to resource-poor health care systems and a dynamic social and political landscape. Since the scale-up of anti-retroviral therapy in SSA in the mid-1990s, more HIV-positive people are surviving into old age [10]. HIV infection itself is an independent risk factor for the development of geriatric syndromes, such as frailty [11]. It is unclear what effect communicable diseases, such as HIV, and limited access to healthcare have had on the health of older adults in SSA.

The World Health Organization calls for the alignment of health systems with the needs of older populations in the Global Strategy Plan on Ageing and Health [12]. This has been echoed on the African continent in the African Union Policy Framework and Plan on Ageing (AU plan) [13]. However, due to limited data on the health and well-being of the aged on the continent, public health systems in SSA have been unable to plan adequately for the needs of ageing populations. National Health Insurance Schemes (NHIS), such as the one implemented in Ghana, aim to provide Universal Health Coverage (UHC) to all, but do not consider the increased burden of age-related health conditions by older adults [14]. National Health Insurance Schemes focus on primary health care as the foundation for UHC, but do not provide coverage for most age-related health conditions such as disabilities, visual and auditory impairments [15]. Less than one in five people over the age of 60 years in SSA receives a pension or benefits from social security [16]. The failure of health systems to address the needs of older adults can lead to catastrophic out-of-pocket expenditure and neglect of treatable age-related health conditions.

The main objective for the proposed scoping review is to identify, explore and map literature on age-related health conditions and associated factors in older adults accessing primary care in sub-Saharan Africa. It is anticipated that the results of a scoping review will inform governments and policymakers of the geriatric health services required by older adults in sub-Saharan Africa and identify gaps for further research. The results will also ensure that health professions educators are aware of the appropriate geriatric competencies that need to be included in local medical curricula.

## Methodology

### Scoping review

This protocol is for a systematic scoping review of literature reporting on age-related health conditions in geriatric populations (i.e. people 60 years and older) in sub-Saharan Africa. A scoping review method was selected as it aims to outline different types of evidence on the area of interest and the gaps for further research. The proposed review will be guided by the methodological framework proposed by Arksey and O’Malley [17]. Thus, the following five steps will be followed in this scoping review: (i) identifying the research question, (ii) identifying relevant studies, (iii) selection of eligible studies, (iv) charting the data, and (v) collating and summarising the results. Quality appraisal will not be done as this review aims to map all research activities in this field.

### Identifying the research question

The main research question is “what are the age-related health conditions reported on in people aged 60 years and older who access primary care services in sub-Saharan Africa?”

The research sub-questions are:

1. What are the different age-related health conditions reported on in people aged 60 years and older in SSA at primary care level?
2. What are the factors associated with age-related conditions in people aged 60 years and older in SSA?
3. How can primary care services address age-related health conditions in people aged 60 years and older in SSA?

This study will use the PEO format (Table 1) to align the study selection with the research question.

**Table 1:** A PEO framework for eligibility of studies

Criteria	Determinants
P-Population	Adults 60 years and older in SSA
E-Exposure	Ageing
O-Outcomes	• Geriatric syndromes
	• Chronic illnesses
	• Functional status
	• Primary healthcare needs

### Identifying relevant studies

A search will be conducted for published and unpublished (grey) literature on the research area in the following electronic databases: Cochrane Library, World of Science, PubMed and WorldCat. Studies published prior to June 2019 that have the keywords or Medical Subject Headings (MeSH) terms “older adults” or “aged”, “primary care” or “health” and “sub-Saharan Africa” will be identified. The search strategy will be piloted to check the appropriateness of keywords and databases. Keywords may be refined to include specific geriatric syndromes such as “dementia”, “falls” and “functional impairment”. A hand search will be also conducted of the references of the included studies and websites such as the World Health Organization (WHO) and the Directory of Research on Ageing in Africa to identify potential relevant literature. Potentially relevant grey literature will be identified through targeted searches of dissertations/theses (ProQuest Dissertations & Theses Global) and conference abstracts (EMBASE Conference Abstracts, Conference Proceedings Citation Index—Science and Social Science & Humanities).

### Selection of eligible studies

Title and abstract screening will be guided by the PEO framework (Table 1). Further eligibility criteria will ensure that the content of the included studies is relevant to the research question.

### Inclusion criteria

For studies to be included, they must meet the following criteria:

- Focus on people or populations aged 60 years or older
- Report on health or primary care services provided to older adults
- Include participants from SSA
- Published prior to June 2019
- Qualitative and quantitative studies

### Exclusion criteria

Studies will be excluded if they have any of the following characteristics.

- Studies that do not include participants or studies from SSA
- Studies looking at geriatric in-patients or specialised services for geriatrics
- Studies where full-text article could not be obtained

The search strategy will be piloted to check the appropriateness of keywords and databases. The electronic database search will be recorded in a table. A draft is provided in Table 2.

**Table 2:** Electronic database searches

Date of search	Electronic database	Keywords searched	Number of studies retrieved	Number of studies selected
01/07/2019	World of Science	“older adults” or “aged”, AND “primary care” or “health” AND “sub-Saharan Africa”		

All eligible articles will be uploaded into Endnote X9 software, and duplicates identified and removed. Between July and August 2019, the authors plan to conduct title and abstract screening of all eligible articles to determine whether the study should be included in the review or not. All attempts will be made to obtain full texts of selected articles, by searching the web, engaging with the UKZN librarian or contacting the author if necessary. Both authors will conduct full-text screening of the selected studies. A third reviewer will be employed if there are significant discrepancies that cannot be resolved by discussion and consensus. The degree of agreement between reviewers will be calculated and reported.

The selection process will follow the recommendations in the Preferred Reporting Items for Systematic Reviews and Meta-Analyses Extension for Scoping Reviews (PRISMA-ScR) checklist [18] and be mapped using the PRISMA-P chart [19]. Selection of studies to be included in the review is expected to be completed within 6 weeks.

### Charting the data

A data charting form will be used to electronically capture relevant information from each included study. This is planned for September 2019. The extracted data will include the following fields (Table 3).

**Table 3:** Data charting form

Author and date	
Title of study	
Publication	
Aim of study	
Study setting	
Study population	
Sampling method	
Study design	
Data collection methods	
Data analysis	
Conclusion	
Outcome	Study findings relevant to study objectives
Most relevant findings	Identification of age-related health condition, and associated factors, primary care service provision for age-related health condition
Comment	

### Collating, summarising and reporting the results

A narrative report will be produced to summarise the extracted data around the following outcomes: region of study, category of age-related health condition, prevalence, associated factors for age-related health conditions and primary care services for older adults. These results will be described in relation to the research question and in the context of the overall study purpose. Gap identification will detect areas, such as countries in SSA that lack data on the health of older adults, and if there is a paucity of data on significant geriatric health conditions.

## Discussion

The proposed scoping review aims to identify and describe age-related health conditions in geriatric populations in sub-Saharan Africa. It will also highlight gaps regarding knowledge of geriatric health in SSA.

This review will be the first part of a study to develop guidelines for health professions education in geriatric primary care. An understanding of the primary healthcare needs of older adults in SSA will assist health professions educators to design and implement age-friendly medical training programmes. This will capacitate medical graduates to suitably care for older adults at primary care level. This review also has the potential to create greater awareness into the growing health care needs of older adults in the region and will provide evidence to assist health policymakers and stakeholders to address the needs of this vulnerable population.

A limitation of this review is that it may omit studies that include participants of all ages, including those aged 60 years and older. Studies that define older adults as 50 years and older may be omitted from the review if data on people aged 60 years and older cannot be isolated from the results. This may result in the exclusion of important studies such as the World Health Organization’s multi-country Study on global AGEing and adult health (WHO SAGE) [20]. Also, since the quality of the studies will not be assessed, the reliability of data extracted from selected studies cannot be commented on.

## Data Availability

All data generated or analysed during this study will be included in the published scoping review article.

## Abbreviations

AU: African Union
HIV: Human immunodeficiency virus
MeSH: Medical Subject Headings
NHIS: National Health Insurance Schemes
PRISMA: Preferred Reporting Items for Systematic Reviews and Meta-Analyses
PRISMA-ScR: Preferred Reporting Items for Systematic Reviews and Meta-Analyses Extension for Scoping Reviews
SSA: Sub-Saharan Africa
UHC: Universal Health Coverage
WHO: World Health Organization

## References

[1] Aboderin IA, Beard JR (2015). Older people's health in sub-Saharan Africa. Lancet.

[2] Kowal Paul R., Wolfson Lara J., Dowd John E. (2000). Creating a minimum data set on ageing in sub-Saharan Africa. Southern African Journal of Gerontology.

[3] Aboderin I (2010). Understanding and advancing the health of older populations in sub-Saharan Africa: policy perspectives and evidence needs. Public Health Rev.

[4] Frost L, Liddie Navarro A, Lynch M, Campbell M, Orcutt M, Trelfa A (2015). Care of the elderly: survey of teaching in an aging sub-Saharan Africa. Gerontol Geriatr Educ.

[5] Dotchin CL, Akinyemi RO, Gray WK, Walker RW (2013). Geriatric medicine: services and training in Africa. Age Ageing.

[6] Chatterji S, Byles J, Cutler D, Seeman T, Verdes E (2015). Health, functioning, and disability in older adults—present status and future implications. Lancet.

[7] Inouye SK, Studenski S, Tinetti ME, Kuchel GA (2007). Geriatric syndromes: clinical, research, and policy implications of a core geriatric concept: (see editorial comments by Dr. William Hazzard on pp 794–796). J Am Geriatr Soc.

[8] Prince M, Acosta D, Chiu H, Scazufca M, Varghese M (2003). Dementia diagnosis in developing countries: a cross-cultural validation study. Lancet.

[9] Tinetti ME, Inouye SK, Gill TM, Doucette JT (1995). Shared risk factors for falls, incontinence, and functional dependence: unifying the approach to geriatric syndromes. Jama..

[10] Deeks SG, Lewin SR, Havlir DV (2013). The end of AIDS: HIV infection as a chronic disease. Lancet.

[11] Pathai S, Gilbert C, Weiss HA, Cook C, Wood R, Bekker L-G (2013). Frailty in HIV-infected adults in South Africa. J Acquir Immune Defic Syndr.

[12] Beard JR, Officer A, de Carvalho IA, Sadana R, Pot AM, Michel J-P (2016). The world report on ageing and health: a policy framework for healthy ageing. Lancet.

[13] Union A. Africa Health Strategy 2016–2030. p. 2016.

[14] Peltzer K, Williams JS, Kowal P, Negin J, Snodgrass JJ, Yawson A (2014). Universal health coverage in emerging economies: findings on health care utilization by older adults in China, Ghana, India, Mexico, the Russian Federation, and South Africa. Glob Health Action.

[15] McIntyre D, Garshong B, Mtei G, Meheus F, Thiede M, Akazili J (2008). Beyond fragmentation and towards universal coverage: insights from Ghana, South Africa and the United Republic of Tanzania. Bull World Health Organ.

[16] Bloom DE, Canning D, Lubet AJD. Global population aging: facts, challenges, solutions & perspectives. Daedalus. 2015;144(2):80–92.

[17] Arksey H, O'Malley L (2005). Scoping studies: towards a methodological framework. Int J Soc Res Methodol.

[18] Tricco Andrea C., Lillie Erin, Zarin Wasifa, O'Brien Kelly K., Colquhoun Heather, Levac Danielle, Moher David, Peters Micah D.J., Horsley Tanya, Weeks Laura, Hempel Susanne, Akl Elie A., Chang Christine, McGowan Jessie, Stewart Lesley, Hartling Lisa, Aldcroft Adrian, Wilson Michael G., Garritty Chantelle, Lewin Simon, Godfrey Christina M., Macdonald Marilyn T., Langlois Etienne V., Soares-Weiser Karla, Moriarty Jo, Clifford Tammy, Tunçalp Özge, Straus Sharon E. (2018). PRISMA Extension for Scoping Reviews (PRISMA-ScR): Checklist and Explanation. Annals of Internal Medicine.

[19] Moher D, Shamseer L, Clarke M, Ghersi D, Liberati A, Petticrew M (2015). Preferred reporting items for systematic review and meta-analysis protocols (PRISMA-P) 2015 statement. Syst Rev.

[20] Arokiasamy P, Kowal P, Capistrant BD, Gildner TE, Thiele E, Biritwum RB, Yawson AE, Mensah G, Maximova T, Wu F, Guo Y (2017). Chronic noncommunicable diseases in 6 low-and middle-income countries: findings from wave 1 of the World Health Organization's study on global Ageing and adult health (SAGE). Am J Epidemiol.

